# Stakeholder involvement in the development of trial material for a clinical trial

**DOI:** 10.1111/hex.13181

**Published:** 2020-12-14

**Authors:** Jacqueline Rix, Jonathan Branney, Alexander C. Breen, Philip Sewell, Sharon Docherty

**Affiliations:** ^1^ Department of Design and Engineering Faculty of Science and Technology Bournemouth University Poole UK; ^2^ Centre for Biomechanics Research AECC University College Bournemouth UK; ^3^ Department of Nursing Science Faculty of Health and Social Science Bournemouth University Poole UK; ^4^ Department of Medical Science & Public Health Faculty of Health & Social Sciences Bournemouth University Poole UK

**Keywords:** clinical trial, informed consent, Patient and Public Involvement, Research Collaboration, Stakeholder Involvement

## Abstract

**Background:**

Stakeholder involvement includes not just patients and public, but also those delivering treatment for example clinicians and students. Each stakeholder brings unique experiences to the process. The aim of this stakeholder exercise was to explore readability and understanding of the trial material for the future trial to be conducted by the authors: Biomechanical Effects of Manual Therapy—A Feasibility Study.

**Design:**

Volunteers from identified stakeholder groups were provided with trial material which included the information sheet, consent form, questionnaires and home management booklet. They provided feedback on content (readability, understanding) and style (font, layout). An additional document was provided with genres of pictures to choose the most appropriate style to be used in the booklet.

Readability formulas were used to calculate reading age before and after feedback to objectively measure ease of reading.

**Results:**

The public group provided a layperson's perspective to clarify the information sheet for patients, whereas practitioner and intern groups indicated where information could be clarified. The reading age of all documentation decreased following feedback; however, templated sections of the documentation did not. The majority (87%) of volunteers chose coloured classic cartoons for the booklet.

**Conclusion:**

This process highlighted the importance of involving different stakeholder groups in the development of research materials as each group made a unique contribution. Readability and understanding of the trial material were improved, feeding back into the consent process contributing towards fully informed consent.

**Patient or Public Contribution:**

Public helped develop materials for a future trial but not with manuscript preparation.

## 1 BACKGROUND

It is suggested that a large quantity of health research in the United Kingdom is avoidably wasted.^1^ Some of this research waste has been attributed to failure to publish findings; unclear reporting of findings; and failure of new research studies to systematically review previous work in similar fields, resulting in unnecessary replication.^2^, ^3^ Patients and members of the public have an interest in and role to play in research waste reduction.^2^

Involving and collaborating with patients and members of the public can improve study design, methods and relevance of research.^4^, ^5^ Together with researchers, they can be involved in decisions regarding how studies are prioritized, designed and conducted.^2^ This improves research by making it more relevant to the patient.^2^, ^6^ Patients and members of the public can also bring different perspectives from those who are conducting the research, such as a lay person's perspective, as well as the lived experience of the condition or caring for someone with the condition.^5^ Patient and public involvement (PPI) may be used to aid development of trial material for patients which may have an impact in the recruitment and retention of trial participants,^4^, ^6^, ^7^ as well as the consent process.^8^

Stakeholder involvement is inclusive participation of all stakeholders in health‐care research, from the grass roots student population to clinicians and from the public to patient. Each volunteer group brings unique experiences, knowledge and skill sets to the development of health‐care research creating an authentic partnership of self‐identified volunteers working towards a common goal.^9^

While much of the research carried out and published in the area of stakeholder involvement is centred around PPI, there is only a small amount of literature extending the sphere of collaboration to professional members of the health‐care community who may have an impact on, or be impacted by, the subject under investigation.^9^ It is suggested that health‐care professionals may provide personal insight into clinical trial development, as well as a breadth of knowledge relating to clinical trial design and interventions.^10^ Equally, health‐care professionals may provide insight into whether the trial would be practical, useful or usable in a particular setting.^11^

By combining the public and patient experience and perspective, together with the knowledge and personal insight of a health‐care professional, a well‐rounded unique view of the study can be obtained and can enhance the development of a clinical trial.^10^ Equally, involving patients and members of the public, together with health‐care professionals in research is vital as they are the end‐users.^10^

While the primary goal of health‐care research is to generate new knowledge, according to the Declaration of Helsinki, this cannot take precedence over the interests and rights of human research participants.^12^ Informed consent is the cornerstone of ethical research.^13^ In health‐care research, when informed consent is given, it indicates an individual has made a fully informed and voluntary decision to take part in the research trial. Therefore, the onus lies with the team conducting the trial to support the consent process by providing the participant with adequate details of the trial reasoning and procedures. This information should provide potential participants with all the materials they require to make an informed decision about their participation in the trial.^14^ For example, the purpose of the research; potential benefits and risks; the right to refuse or withdraw; and treatment alternatives.^12^ However, the quantity of information given can overwhelm participants^15^, ^16^ and may lead to a participant's lack of understanding of key aspects of the trial which could be considered crucial information to those who are considering their participation.^17^, ^18^, ^19^ This may be due to the information being supplied in a complex way, not designed to support a participant's informed decision process, or using language which is better suited to a medical professional rather than a trial participant or lay person.^19^, ^20^

An additional complexity in the consent process occurs when potential participants lack adequate literacy to be able to read and understand the information sheet and consent form. The National Literacy Trust^21^ indicates that the 16.4% of adults in England have ‘very poor literacy skills’ and are defined as functionally illiterate (a reading age at or below the average 11‐year‐old). Up to 74% of studies relating to informed consent and participant comprehension of research information do not assess participant comprehension, which may contribute to a lack of participant understanding.^18^

One way to assess comprehension of text is to use readability formulas. These formulas generate automated numerical estimates of readability of a text. The readability formulas focus on the average number of syllables in a word (word length) and the number of words in a sentence (sentence length). Three readability formulas considered objective measures of text comprehension are:

- The Flesch Reading Ease Formula^22^ will output a number ranging from 0‐100, the higher score indicates easier reading. A score of 90‐100 can be understood by an average fifth‐grade student (10‐11 year old); 60‐70 can be understood by an average eighth or ninth grade student (13‐15 year old); 0‐30 can be understood by an average university student (18‐21 year old).
- The Flesch‐Kincaid Grade Level^23^ indicates a school grade level (USA) which the average student in that grade would be able to read.
- The Gunning Fog Formula^24^ is a scale that indicates syllable and sentence length. A Fog score of 5 is readable, 10 is hard, 15 is difficult and 20 is very difficult.

However, the formulas may not be an appropriate predictor of comprehension for short question surveys or questionnaires.^25^

In addition, many studies do not consider additional impacts on readability such as layout, appearance, font size, and use of diagrams or pictures.^26^ According to the British Dyslexia Association,^27^ up to 10% of the British population has some degree of dyslexia. As such, Dyslexia Guidelines should be considered when designing an information sheet or consent form for ease of readability.

The use of pictures in health‐care booklets or information sheets enhances engagement of patients^28^ and can facilitate comprehension of the written word.^29^ Patients have indicated that booklets with pictures are easier to read than text alone, even when the written text is identical.^28^, ^30^ Additionally, photo or picture elicitation has been used in social science research in a variety of ways.^31^ Images can elicit moods or feelings distinct from written text.^31^, ^32^ As such, consideration of the photograph or picture genre can aid understanding or create misunderstanding depending on the genre choice.^32^ In the case of health‐care research, this could mean the difference between the participant feeling informed and reassured, versus feeling fearful or that their condition is not being taken seriously.

This Stakeholder Involvement Process examined the material for the trial entitled: Biomechanical Effects of Manual Therapy – A Feasibility Study. The trial material examined in this process included the Participant Information Sheet, Participant Consent Form, the trial questionnaires, and the home management booklet for low back pain. This process included stakeholders chosen for their unique experience and expertise which would encompass feedback from a layperson's, as well as, practitioner's perspective. The layperson brought their experience of the lived experience of low back pain, while practitioners brought their wealth of knowledge in the subject area, and their years of experience in practice. Intern students brought their theoretical knowledge, but more importantly their experience of working in the environment where the future trial will take place. The primary aim of this process was to improve the comprehension (through improvements in readability and understanding) of the trial materials for the future participants, equally to contribute towards the development of a comprehensive information sheet. Both contribute towards informed consent for the future trial participants.

## 2 METHOD

### 2.1 Ethics

A Stakeholder Involvement Process is a collaborative process, and not considered research by the NHS.^33^ Following completion of the HRA NHS Review decision tool^34^ and under the advice of local ethical guidelines, ethical approval was not required.

### 2.2 Volunteer Recruitment

Five volunteers were sought from each the following groups, each group was recruited via separate advertising strategies. All advertising and recruitment material specified that taking part was voluntary, unfortunately the project did not have a budget to reimburse volunteers.

- Members of the public who have experienced low back pain were recruited via the university public and patient partnership. It was hoped that the public group would be able to give feedback from a lay person's perspective, which would be helpful in ensuring the potential trial participants would be able to read and understand what is involved with taking part in the trial.
- Registered chiropractic clinicians who have been in practice for at least two years were recruited via an advertising email. Emails were sent to Chiropractic Institution tutors who met the relevant criteria. It was expected that the practitioner group would be able to identify areas of missing information or pertinent information not highlighted sufficiently.
- Chiropractic intern students (final year chiropractic student clinicians) were recruited via an advertising email. Emails were sent via the Chiropractic Institution tutors. It was expected that the intern group would be able to give feedback from their strength in theoretical knowledge. Equally, the intern students work in the same building where the trial will take place, as such it was expected their feedback may highlight practical considerations related to carrying out the trial.

The collaboration process was carried out from the beginning of April 2019 until the end of June 2019.

### 2.3 Collaboration process

All volunteers were sent the trial documentation, either as an electronic version (by email) or as a paper copy, depending on their preference or level of IT skills. These included the information sheet, consent form, questionnaires, and home management booklet. An additional document which included different genres of pictures for potential use within the home management booklet was also provided. All documents sent to volunteers were single spaced, using the font Calibri in a size 11 (Microsoft® Word for Microsoft Office 365, USA). Volunteers were asked to provide feedback on the documentation, this could be completed either as ‘tracked changes’ in a Microsoft Word Document or as changes made on a paper copy. The individual documents and requested feedback are outlined below.

### 2.4 Participant information sheet

The information sheet underpins the participant understanding of the trial, what the purpose of the trial is, what their role in the trial will be, withdrawal process and data management. The Health Research Authority template and the university template was used to complete the information sheet.^35^

Feedback was requested on:

- Content:
  - How easy was the wording of the information sheet to read?
  - Was the information sheet clear and easy to understand?
- Style:
  - Was the information sheet easy to read, specifically looking at style layout, font, font size and line spacing)?
- Were there any additional changes to the document that volunteers would like to add? These did not necessarily have to be related to readability and understanding. Practitioners and intern students were asked if they felt there was any missing information or additional information which may be helpful to a participant.

### 2.5 Participant Consent Form

The consent form is a signed agreement between the researcher and the participant indicating that the participant has read and understood the information sheet and is happy to be a part of the trial. The Health Research Authority template and university template was used to complete the consent form.

Feedback was requested on.

- Content
- How easy was the wording of the consent form to read?
- Was the consent form clear and easy to understand?
  - Style
- Was the consent form easy to read, specifically looking at style (layout, font, font size and line spacing)?

### 2.6 The questionnaires

Two questionnaires will be used in the trial, the Bournemouth Questionnaire^36^ and the Roland Morris Disability Questionnaire.^37^ These questionnaires are used as patient‐reported outcomes measures.^38^ As these are validated, the questions cannot be changed without influencing the validity of the questionnaires. Equally, the readability formulas are not very accurate for short questions.^25^

For these reasons, feedback was only requested on style:

- Were the questionnaires easy to read, specifically looking at style (layout, font, font size and line spacing)?

### 2.7 The Home Management Booklet

The evidence informed home management booklet was developed and compiled by the research team from recent published guidelines relating to non‐invasive treatment of acute low back pain. Volunteers were sent the wording of the booklet, without illustrations.

Feedback was requested on:

- Content:
- How easy was the wording of the home management booklet to read?
- Was the home management booklet clear and easy to understand?
- Style:
- Was the home management booklet easy to read, specifically looking at style (layout, font, font size and line spacing)?

**Figure 1:**
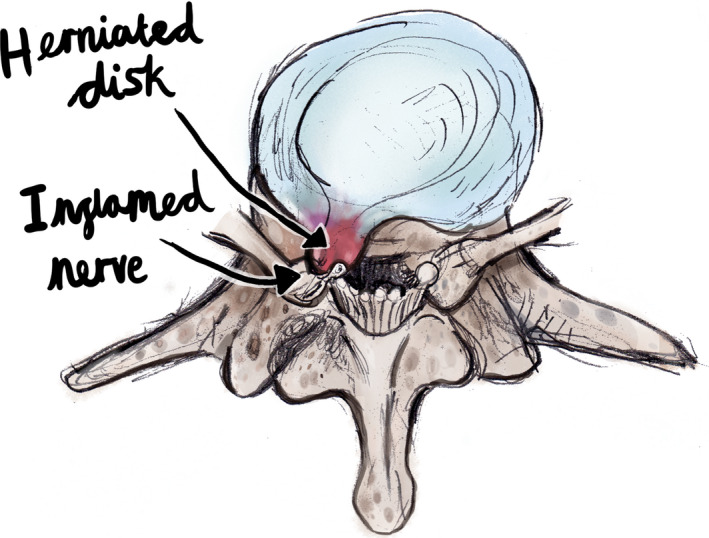
In the category of anatomy of the back, an example of a coloured anatomically correct anime (detailed)

**Figure 2:**
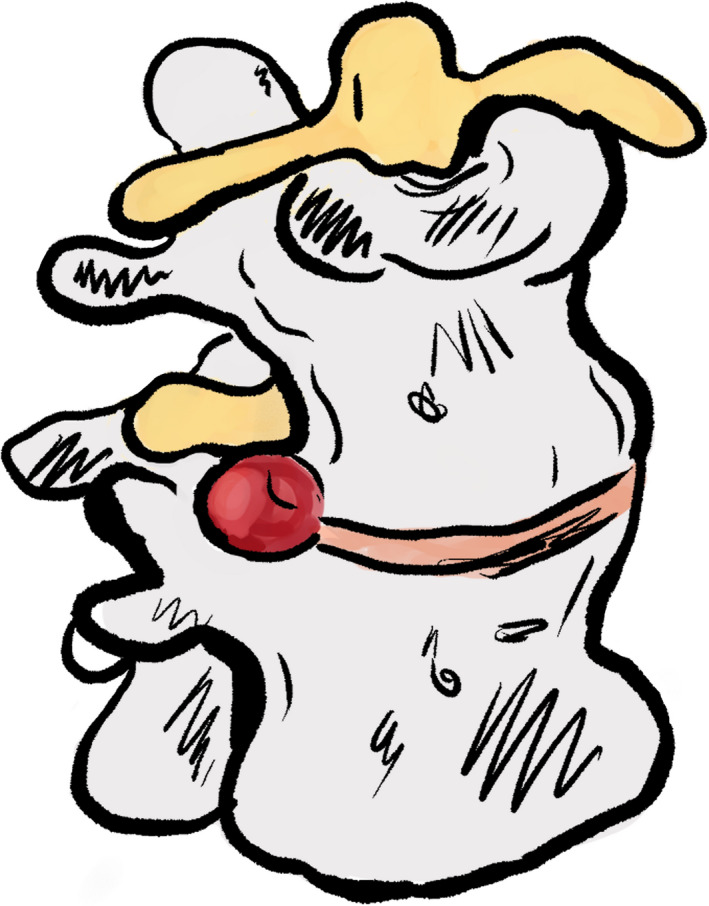
In the category of anatomy of the back, an example of a coloured classic cartoon

**Figure 3:**
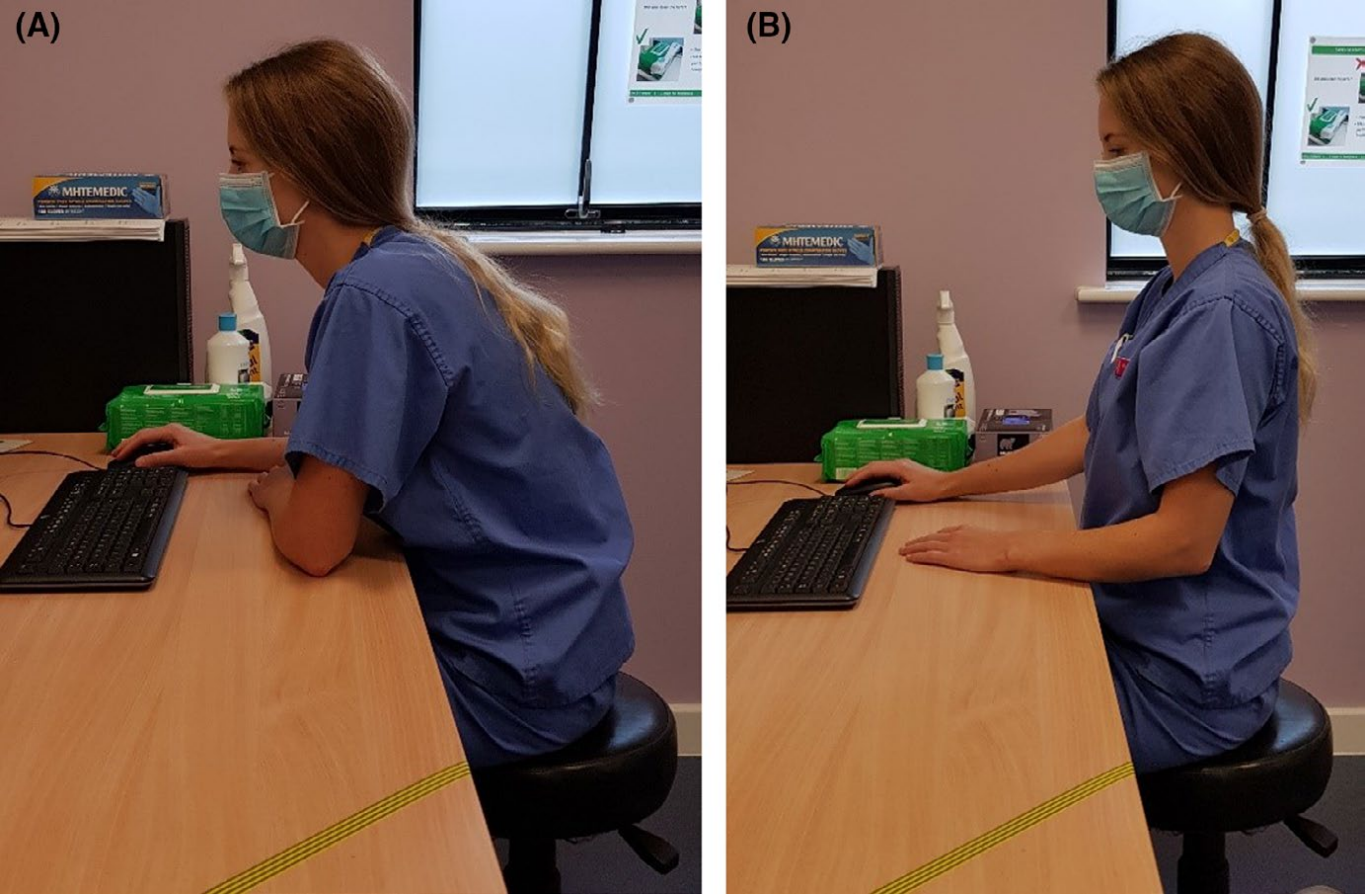
In the category of posture and ergonomics, an example of a photograph

**Figure 4:**
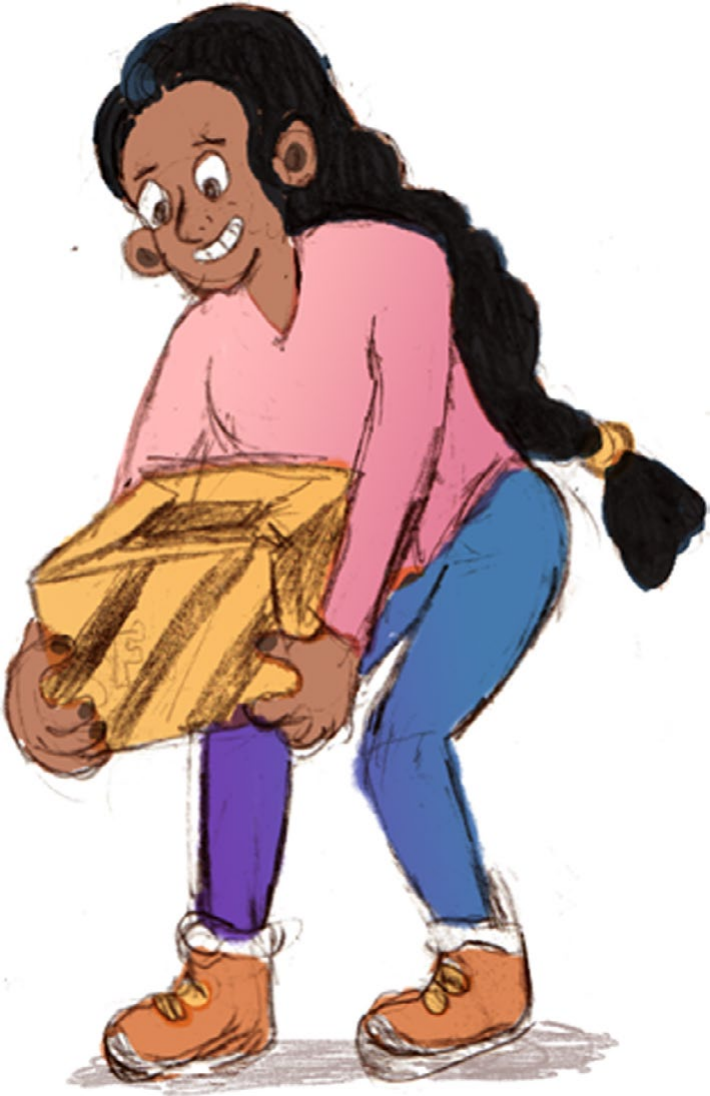
In the category of posture and ergonomics, an example of a coloured classic cartoon

**Figure 5:**
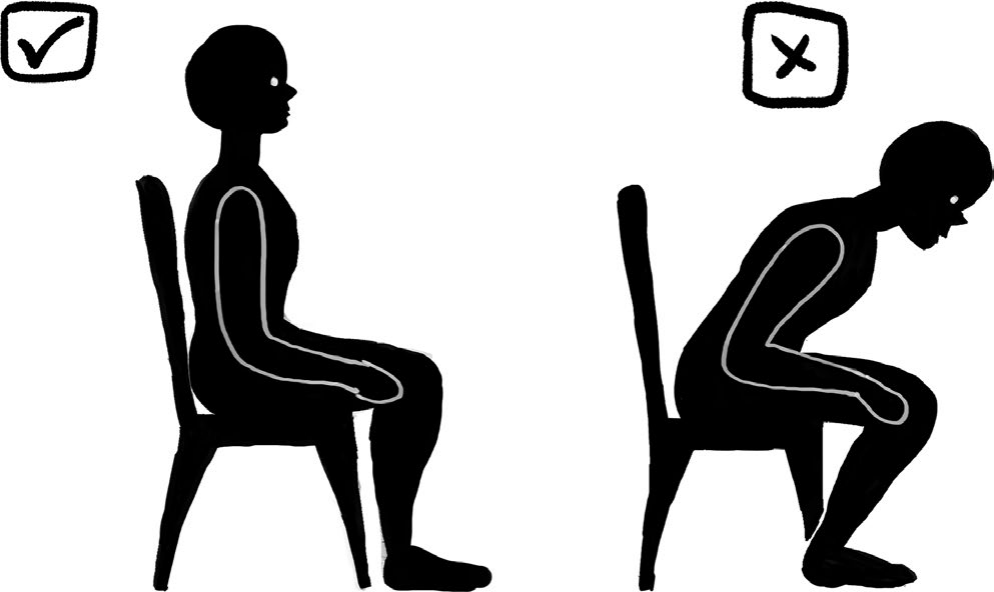
In the category of posture and ergonomics, an example of a black and white stick figure

A document which included different genres of pictures was also sent. Volunteers were asked to select the picture which gave the feeling of reassurance, without giving the feeling that the condition of the patient was not being taken seriously. Pictures were divided into two categories, anatomy of the back, and postural and ergonomic pictures. Pictures were organized from most detailed, to least detailed (see Table 1). Volunteers were supplied with an example of each genre; however, due to copyright, not all pictures could be included in this article. In the category of anatomical pictures, examples of a coloured anatomically correct anime can be seen in Figure 1, and a classic coloured cartoon can be seen in Figure 2. In the category of postural pictures, examples of a photograph can be seen in Figure 3, a coloured classic cartoon can be seen in Figure 4, and a black and white stick figure can be seen in Figure 5. Volunteers were asked to choose one picture from each category.

**Table 1:** Picture genres to be used in the Home Management Booklet (listed from most detailed picture genre to least detailed picture genre for each category). The picture genres were provided to volunteers for each category

Categories	Picture Genres	
Anatomy of the back	Coloured anatomically correct illustration (detailed) Coloured anatomically correct anime Coloured classic cartoon (detailed cartoon) Black and white classic cartoon (detailed cartoon)	Most detailed 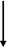 Least detailed
Posture and ergonomics	Photograph Coloured anime Black and white anime Coloured classic cartoon (detailed cartoon) Black and white classic cartoon (detailed cartoon) Simple black and white diagram Black and white stick figure.	Most detailed 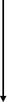 Least detailed

### 2.8 Feedback

A thematic framework analysis of the feedback was carried out.^39^ This is a systematic approach to analysing qualitative data, where commonalities and differences are identified.^40^ Its defining feature is the development of a matrix whereby rows (in this case, each volunteers feedback) and columns (themes) provide a structure in which to systematically manage data and analyse it by theme and individual.^39^, ^40^ The matrix allowed the researcher to organize the data into the three groups to analyse common themes or common aspects of feedback highlighted by each group.^39^

Feedback was combined with the original documents into one Microsoft Word Document (Microsoft® Word for Microsoft Office 365, USA), using ‘tracked changes’. The primary researcher made changes to the original documents accordingly. Changes which reduced the sentence length and number of syllables in the wording were made as this may reduce reading age and increase understanding of the documents. For layout, font, font size and spacing, all feedback and comments were considered together with the dyslexia guidelines.^27^ Regarding the pictures, all feedback was collated and the genre which was chosen most frequently was used for the home management booklet. All modified documents were sent to the remaining researchers for feedback and discussion.

### 2.9 Readability formulas

All documents were tested using the readability formulas of Flesch Reading Ease Formula, Flesch‐Kincaid Grade Level, Gunning Fog Formula,^41^ before and after consultation.

## 3 RESULTS

### 3.1 Demographic data

A total of fifteen volunteers took part in the process:

- Members of the public: 4 females, 1 male; age range 38‐73 years.
- Registered Chiropractic Clinicians: 3 females, 2 males; age range 31‐47 years.
- Chiropractic Intern Students: 4 females, 1 male; age range 23‐31 years.

### 3.2 Feedback

In general, the public group provided more feedback than the remaining two groups. The thematic framework analysis identified themes within the feedback. Once the thematic matrix was complete, common within group themes were identified (see Table 2).

**Table 2:** Thematic framework analysis of common themes within groups and between groups related to content (readability and understanding) and style

Group Theme	Public	Chiropractic Practitioners	Chiropractic Interns
Length of Participant Information Sheet:	It was felt by four of the public volunteers that the information sheet was too long, they questioned if all the information outlined was necessary.	No comments	No comments
Length of paragraphs and sentences in the Participant Information Sheet:	Some feedback was given for sentence length, such as ‘The consent process is a bit wordy, can you simplify it?’	Some feedback was given for paragraph length, such as ‘Split this paragraph here in two paragraphs, it's really long otherwise’	No comments
Use of language (and medical terms) in all documents:	All five volunteers identified language which they did not understand, such as ‘can the withdrawal section be made simpler? Instead of withdrawal, can you just say stop participating’; ‘I don't know what ‘randomisation’ meant, I had to look it up’; ‘what do you mean by an ‘incidental finding’ on x‐ray?’. The group also felt that the consent form contained ‘big words’ and felt these needed to be simplified.	No comments	No comments
Insufficient information in the Participant Information Sheet:	No Comments	Some feedback was given regarding eligibility criteria. It was felt inclusion and exclusion criteria could be elaborated upon. Some feedback was given relating to the incidental x‐ray findings as it was felt these could be listed. Alternative treatments available for the same condition were not outlined in the document.	Some feedback was given regarding the possible incidental findings on an x‐ray. It was felt that this could be explained better or a list of possible incidental findings being provided.
Sequence and Flow of the Participant Information Sheet:	No comments	No comment	Four of the chiropractic Interns identified sequence errors in the section related to what the participants would be required to do in the study. Two of the volunteers changed the sequence using ‘tracked changes’ to create a more logical sequence of events.
The addition of pictures in the Participant Information Sheet:	Some feedback was given by the public group to add photos or pictures of ‘scary equipment’ to give the participants an idea of what to expect particularly relating to fluoroscopy.	No comment	No comment
Data Management sections in the Participant Information Sheet and Participant Consent Form:	The public volunteers struggled to understand both documents. In summary, ‘I don't understand any of this, basically will you keep my data safe’	No comment	No comment
Home Management Booklet	Some feedback was given relating to the hot and cold pack section, can more options be listed or signpost participants to their pharmacy for other options. One participant suggested that I inform participants to speak to their pharmacist before taking any medication for their back pain. One participant recommended when pictures are added to the booklet, that they reflect a diverse population.	No comment	Two of the five volunteers suggested that an exercise and rehabilitation section be added to the booklet.
Font, font size and layout	One comment relating to colour used within documents, ‘it's all so black and white, it makes my eyes sore’. It was suggested that the font was quite small and could be made bigger. One person commented that they liked the font in the Information Sheet, but not in the Home Management Booklet. However, the fonts used were all the same across all documents.	It was suggested that the font spacing be 1.5 spaced to allow the reader to read the document more easily.	No comment

The public group had concerns about the length of the information sheet, in contrast, the intern and practitioner groups provided feedback on where they felt there was insufficient information and what could be added to the information sheet. The public group provided feedback on length of sentences and use of language (particularly medical terms). Equally, they felt that the use of pictures may help manage future participants expectations of the trial. Neither the practitioner group, nor the intern student group identified this in their feedback. The public group provided feedback on the data management section of the information sheet, indicating that this section was difficult to understand. Neither the practitioner group, nor the student intern group identified this in their feedback. The intern group provided feedback on the practicalities of running the future trial in the clinic. Neither the practitioner group, nor the public group identified this in their feedback.

Both the public group and the student intern group provided feedback on the home management booklet, suggesting that further information could be added. The practitioner group did not identify this in their feedback.

The public group and practitioner group provided feedback on structure (font and layout), the student intern group did not. Feedback included making the font larger, as well as increasing the spacing between lines. The public group felt the booklet was very black and white and would prefer the addition of colour.

### 3.3 Picture Feedback

#### 3.3.1 Images of Back Anatomy

Thirteen of the 15 volunteers chose coloured classic cartoons (detailed cartoon):

- Three out of five volunteers in the public group chose coloured classic cartoon (detailed cartoon) (see Figure 2). One participant did not choose a picture, and one participant chose coloured anatomically correct anime (see Figure 1).
- Five out of five chiropractic practitioners, as well as five out of five student interns chose coloured classic cartoon (detailed cartoon) (see Figure 2). Although volunteers were not asked for further feedback, one volunteer commented that the coloured anatomically correct illustration was quite scary to a lay person and could create more anxiety about their low back pain.

#### 3.3.2 Posture and Ergonomics

Again, thirteen volunteers chose coloured classic cartoon (detailed cartoon):

- Three out of five volunteers in the public group chose coloured classic cartoon (detailed cartoon) (see Figure 4). One participant did not choose a picture, and one participant chose stick figures (see Figure 5).
- Five out of five chiropractic practitioners, as well as five out of five student interns chose coloured classic cartoon (detailed cartoon) (see Figure 4).

#### 3.3.3 Readability Scores

Readability scores for the trial documents, information sheet, consent form, and home management booklet were calculated. The scores before and after the Stakeholder Involvement Process can be seen in Table 3.

**Table 3:** Readability Scores of trial documentation before the Stakeholder Involvement Process and after the Stakeholder Involvement Process

Readability Score Document	Flesch Reading Ease		Flesch‐Kincaid Grade Level		Gunning Fog Formula	
	Before	After	Before	After	Before	After
Participant Information Sheet (Whole document)	39.4 (difficult to read)	39.4 (difficult to read)	12.1 (twelfth grade (17 years old))	12.1 (twelfth grade (17 years old))	14 (hard to read)	14 (hard to read)
Participant Information Sheet without Data Management Section	38.0 (difficult to read)	72.0 (13‐15 years old)	12.1 (twelfth grade (17 years old))	7.9 (eighth grade (13‐14 years old))	14 (hard to read)	10.5 (hard to read)
Participant Information Sheet, Data Management Section Only	39.8 (difficult to read)	39.8 (difficult to read)	12.1 (twelfth grade (17 years old))	12.1 (twelfth grade (17 years old))	14 (hard to read)	14 (hard to read)
Participant Consent Form	45.8 (difficult to read)	45.8 (difficult to read)	12.0 (twelfth grade (17 years old))	12.0 (twelfth grade (17 years old))	13.4 (hard to read)	13.4 (hard to read)
Home Management Booklet	70.4 (13‐15 years old)	79.2 (11 years old)	7.7 (eighth grade (13‐14 years old))	5.9 (sixth grade (11 years old))	10.9 (hard to read)	9.4 (fairly easy to read)

## 4 DISCUSSION

The primary aim of this process was to improve the comprehension (through improvements in readability and understanding) of the trial materials for the future participants, as well as to contribute towards the development of a comprehensive information sheet. Both contribute towards informed consent for the future trial participants.

The age range of the public group was 38‐73 years of age, which is slightly older than the age range for the future trial of 18‐65 years of age. The location of the clinic where the trial will be taking place is ranked 113th in the Index of Multiple Deprivation, meaning it is in the top 1% of the most deprived areas in England.^42^ However, the clinic where the trial will be taking place is a private clinic requiring payment for treatment. Equally, this process did not collect socioeconomic status or level of education from the volunteers. A chiropractic student Intern is completing a Masters (UK) and as such is completing a level 7 qualification, equally qualified chiropractic practitioners would have completed a level 7 qualification at the very least. However, the student intern and practitioner groups were included for their clinical expertise, and not their experience of low back pain. The public group contained current clinic patients, which indicates that the public group is representative of the future trial population who will be recruited from the clinic. Ethnic data was not collected from volunteers, which is a weakness of this stakeholder process. There is a growing need for a wider range of voices to be heard in research and trial development, as such stakeholder processes should consider recruitment of under‐represented groups such as Black, Asian and minority ethnic (BAME) populations.

Few studies related to readability and understanding of trial documentation calculate readability scores.^18^ In the UK, the Health Research Authority encourage researchers to calculate readability and to ensure that trial documentation is readable for the average person in the UK. The Flesch Reading Ease Formula and Flesch‐Kincaid Grade Level are valid between the ages of 10 and 16. They correlate with Fry and Simple Measures of Gobbledygook readability formulae,^43^ as well as the Cloze Comprehension Test.^44^ However, readability formulae are not without limitations. Readability scores are calculated by readability formulae which are mathematical calculations based on word length, number of words per sentence and number of syllables per word. The formulae may not be able to tell the difference between a heading, a table or a figure and as these have short sentences this may result in a lower score. Equally, the software program being used may see each full stop as the end of a sentence and may not take into account abbreviations or decimals in numbers which may result in a lower score. Not all multisyllabic words are difficult to understand, for example ‘cucumber’ is not considered difficult to read or difficult to understand.^45^

The readability scores of the information sheet and consent form were higher than the level at which the average United Kingdom person would be able to read comfortably. Even when changes were made to the documentation in response to the Stakeholder Involvement Process, such as sentence length and paragraph length, the readability scores did not decrease. Interestingly, when the data management section was removed from the information sheet, the average readability age of the information sheet before feedback changes were made was 17‐year‐old, once changes were made in response to the feedback the readability improved greatly with a readability age of 13‐14 year. The data management section is predominantly a templated section, which cannot be altered. This was mirrored in the comments from the public group related to the Data Management Section of the information sheet whereby one volunteer stated they did not understand any of it and essentially would like to be reassured that their data are safe. One possible reason for the high reading age in this section may be the use of legal language and jargon related to data management and data protection. Neither the practitioner group, nor the intern group raised comments related to the level of the language in the Data Management section. As the template cannot be altered, this is a limitation of this process. The consent form also largely contains templated wording, which may result in the readability age being so high. The results of this stakeholder process may provide the template authors with incentive to go through a similar process to reduce the reading age of the templates and increase understanding and comprehension.

Readability formulae can only give a reading level, it does not provide feedback on layout (font style, font size, spacing, colour),^45^ style of writing (context and appropriateness),^45^ difficulty of concept,^46^ prior knowledge^46^ or coherence of text.^46^ Nor does it provide feedback on whether the medical language being used is understandable to the reader.^45^ As such, the stakeholder involvement process examined these potential issues.

It was felt by the public group that the information sheet was very long and questioned whether all information was necessary. This is supported in the literature whereby the volume of information provided to participants may exceed their preference.^15^, ^16^ Neither the practitioner group nor the intern group raised comments relating to the length of the information sheet. Conversely, the practitioner group and the intern group suggested additional information could be added to the information sheet.

Use of language (and medical terms) in the information sheet and consent form was a theme of feedback which was common from all five volunteers in the public group. A lack of understanding of medical words may be a barrier to participants understanding the information sheet.^19^ The use of medical terms was not identified in the comments from the practitioner group or intern group, which indicates that they do not feel the language is an issue. This highlights the need for public involvement in the development of trial documentation. The additional information that the practitioner group and intern group felt was required would essentially increase the medical language used. A high level of medical language can increase participant fear^47^ and potentially decrease recruitment. However, taking all comments on board related to additional information and the use of medical language, changes were made to the section relating to ‘incidental examination findings’ in a way that is hoped to decrease participant fear and increase understanding.

The comments related to readability and understanding of the information sheet and consent form begs the question, is full informed consent taking place? Participants should have an opportunity to ask questions before signing the consent form, however by simply asking the participant ‘do you have any questions?’, this may not be sufficient to reveal whether the participant understands the trial or are signing fully informed consent. This Stakeholder Involvement Process revealed that there is potentially a lack of understanding of the information sheet and consent form. To improve patient understanding interventions have been identified, such as person‐to‐person interactions with an extended discussion to complement the information sheet; multimedia interventions (including video presentation of the trial); enhanced consent forms.^48^ Multimedia interventions and enhanced forms can be expensive and time consuming, equally the literature does not reflect that there is an increased understanding. Literature suggests that a standard format information sheet and consent form, together with a meeting with a research team member for an extended discussion can improve understanding and is inexpensive while using minimal resources.^48^ An extended discussion may consist of a thirty‐minute telephone discussion or a two‐hour face‐to‐face counselling session, there is no strict guideline on this to allow the complexity of the trial to guide the amount of time spent with a prospective participant. It is suggested that an extended discussion whereby participants are quizzed or asked to explain their understanding of the trial back to the researcher is beneficial for trial understanding.^49^ A lack of a definitive definition of ‘understanding’ exists in the literature, which can make it difficult to ascertain whether understanding has taken place. What the literature can agree on is that further empirical research is required in the area of informed consent.^49^, ^50^ Reflecting upon feedback from this collaboration process, an extended discussion with participants in the future trial should include the meaning and implications of randomization, what will happen to the participant during the trial, and data management.

Volunteers were also asked if there was anything they would like to add to the information sheet. The public group suggested the use of pictures, particularly relating to ‘*scary’* medical equipment mentioned in the section related to what will happen to the participant during the trial. Additional pictures were added to the information sheet to aid understanding of the process, as well as managing participant expectations of trial procedures. The intern group suggested changes were made to the sequence and flow of the procedure participants will go through during the trial, this will make the future trial process smoother and more logical for the participants. It will also reduce the participant time burden during the trial.

For the home management booklet, the public group were quick to think of other options for home management, particularly related to how to make a hot or cold pack at home. These were incredibly creative and generally were not added to the booklet as it would increase the length of the booklet considerably. Equally, some of the more creative ideas may increase risk of injury. However, the booklet does now suggest that other heat and cold packs can be used. There was a concern that participants may take over the counter pain medication without speaking to their pharmacist. It should be noted that the section relating to medication already urged all participants to consult their pharmacist before starting to take pain medication, as such no further changes were made. The suggestion from the intern group to include rehabilitation exercises was not considered as this may add an additional confounding variable to the trial; however, a link (web address) to the NHS website was provided which does have information on basic stretches for low backpain.

It has been established in the literature that pictures can complement the written word to increase understanding.^29^ Volunteers were asked to choose the genre of pictures which best elicits the feeling of reassurance, and that the condition is being taken seriously. The majority of volunteers chose coloured classic cartoons for the illustrations for the home management booklet. An understanding of why most volunteers chose the same genre is unclear. However, the use of coloured cartoons can be used to entertain and persuade children and adults alike.^51^ Cartoons can cross barriers of culture, age and literacy which can add to the effectiveness of the communication tool. In line with this, pictures for the book were commissioned to reflect a diverse population as recommended by our public consultation group. An example of one of the illustrations can be seen in Figure 4.

Regarding fonts, font size and layout feedback was in line with the Dyslexia Guidelines.^27^ As a result of the feedback, the font remained as Calibri, a standard Microsoft Word font which is sans serif which makes the font more readable. Italics and underlining were removed, with bold being used for emphasis. Font size was increased from 11 to 12, letter spacing was increased by 20% and line spacing was adjusted to 1.5. The paper used was thicker to ensure that wording on the back of the page cannot be seen through the page.

The altered trial material was not sent back to the volunteers for further feedback. This was largely due to time constraints to this doctoral project, which is not an uncommon issue related to doctoral research.^46^ The altered material was however viewed by the research team for further feedback. The altered material was submitted, together with an ethical application for the future trial to a NHS Research Ethics Committee, which included lay members.

Volunteers were not paid for their time due to budget limitations. This is considered poor practice; however, budgetary limitations are not uncommon in doctoral research and can be a limitation in carrying out stakeholder involvement processes.^52^ Future studies should consider building in a stakeholder process into the proposal and budget calculations of a study.

## 5 CONCLUSION

The Stakeholder Involvement Process was an invaluable exercise that aided the development of the trial documentation. Each group of volunteers made a unique contribution to the study design, the readability and understanding of trial documentation, and the development of the home management booklet. This in turn feeds back into the informed consent process contributing towards fully informed consent by participants in the future trial.

## 6 CONFLICT OF INTEREST STATEMENT

The authors declare that they have no conflict of interest.

## AUTHORS’ CONTRIBUTION

All authors have made a substantial contribution to conception and design. JR carried out the collaborative process, collation of feedback and alteration of trial material. All authors were involved in drafting and revising the manuscript. All authors have given final approval of the version to be published. All authors agree to be accountable for all aspects of this work.

## Funding information

This forms part of a PhD match funded by Bournemouth University and AECC University College.

## Data Availability

Data sharing is not applicable to this article as no new data were created or analysed in this study.
